# Novel pathway for mutagenic tautomerization of classical А∙Т DNA base pairs *via* sequential proton transfer through quasi-orthogonal transition states: A QM/QTAIM investigation

**DOI:** 10.1371/journal.pone.0199044

**Published:** 2018-06-27

**Authors:** Ol’ha O. Brovarets’, Kostiantyn S. Tsiupa, Dmytro M. Hovorun

**Affiliations:** 1 Department of Molecular and Quantum Biophysics, Institute of Molecular Biology and Genetics, National Academy of Sciences of Ukraine, Kyiv, Ukraine; 2 Department of Molecular Biotechnology and Bioinformatics, Institute of High Technologies, Taras Shevchenko National University of Kyiv, Kyiv, Ukraine; University of Calgary, CANADA

## Abstract

In this paper we have theoretically predicted a novel pathway for the mutagenic tautomerization of the classical A∙T DNA base pairs in the free state, the Watson-Crick A·Т(WC), reverse Watson-Crick A·Т(rWC), Hoogsteen A·Т(H) and reverse Hoogsteen A·Т(rH) pairs, *via* sequential proton transfer accompanied by a significant change in the mutual orientation of the bases. Quantum-mechanical (QM) calculations were performed at the MP2/aug-cc-pVDZ//B3LYP/6-311++G(d,p) level in vacuum phase, along with Bader’s quantum theory of Atoms in Molecules (QTAIM). These processes involve transition states (TSs) with quasi-orthogonal structures (symmetry C_1_), which are highly polar, tight ion pairs (A^-^, N6H_2_-deprotonated)∙(T^+^, O4/O2-protonated). Gibbs free energies of activation for the A∙T(WC) / A∙T(rWC) ↔ A*∙Т(rw_WC_) / A*∙Т(w_WC_) tautomeric transitions (~43.5 kcal∙mol^-1^) are lower than for the A∙T(H) / A∙T(rH) ↔ A*_N7_∙Т(rw_H_) / A*_N7_∙Т(w_H_) tautomerisations (~53.0 kcal∙mol^-1^) (rare tautomers are marked by an asterisk; w—wobble configured tautomerisation products). The (T)N3^+^H⋯N1^-^(A), (T)O4^+^H⋯N1^-^(A) / (T)N3^+^H⋯N1^-^(A) and (T)O2^+^H⋯N1^-^(A) H-bonds are found in the transition states TS^A-·T+^_A·T(WC)↔A*·T(rwWC)_ / TS^A-·T+^_A·T(rWC)↔A*·T(wWC)_. However, in the transition state TS^A-·T+^_A·Т(H)↔A*N7·T(rwH)_ / TS^A-·T+^_A·Т(rH)↔A*N7·T(wH)_, the (T)N3^+^H⋯N7^-^(A), (T)O4^+^H⋯N7^-^(A) / (T)N3^+^H⋯N7^-^(A) and (T)O2^+^H⋯N7^-^(A) H-bonds are supplemented by the attractive (T)O4^+^/O2^+^⋯N6^-^(A) van der Waals contacts. It was demonstrated that the products of the tautomerization of the classical A∙T DNA base pairs—A*∙Т(rw_WC_), A*_N7_∙Т(rw_H_) and A*_N7_∙Т(w_H_) (symmetry C_s_)–further transform *via* double proton transfer into the energetically favorable wobble A∙T*(rw_WC_), A∙T*(rw_H_) and A∙T*_O2_(w_H_) base mispairs (symmetry C_s_).

## Introduction

Investigation of microstructural mechanisms for mutagenic tautomerization of the Watson-Crick DNA base pairs occupies an important place in molecular biophysics and molecular biology, enabling an understanding of the nature of genome instability [1–5]. This follows from the ‘rare tautomer hypothesis’ proposed by Watson and Crick [1] shortly after they established the spatial architecture of DNA [2]. However, achievements in this area remain rather modest despite its long history [6], encouraging further research in this direction.

Löwdin [3, 4] first proposed that the electronic structure of the Watson-Crick (WC) DNA base pairs A∙T(WC) and G∙C(WC) permits their transition into the high-energy tautomerized states A*∙T*(L) and G*∙C*(L), now called Löwdin (L) base pairs. Here and henceforth, rare (in particular mutagenic) tautomers are marked with an asterisk and differ from each other by the location of a particular proton: in the A* rare tautomer proton bonds to N1 nitrogen atom, A*_N7_ –to N7 nitrogen atom; T*–to O4 oxygen atom and T*_O2_ –to O2 oxygen atom. Löwdin proposed that the A∙T(WC)↔A*∙T*(L) and G∙C(WC)↔G*∙C*(L) transitions occur by double proton transfer (DPT) along neighboring intermolecular hydrogen (H) bonds *via* proton tunneling. These ideas have been prominent in the field of quantum biology and attracted much theoretical study of the mechanisms of spontaneous transitions and transversions arising during DNA replication [7–14].

Recently, it has become clear that Löwdin’s mechanism does not provide the generation of sufficiently long-lived mutagenic tautomers of the DNA bases, which escape from the replicative DNA-polymerase transforming into their canonical tautomeric forms. The root cause of this observation is the absence of the reverse barrier of tautomerization ΔΔG in the A∙T(WC) DNA base pair and its small value in comparison with *kT* (0.62 kcal∙mol^-1^ under normal conditions) for the G∙C(WC) DNA base pair [8, 9, 15–18].

In previous papers [19–27] we proposed an alternative mechanism for mutagenic tautomerization of the A∙T(WC) and G∙C(WC) base pairs into the corresponding wobble base mispairs and *vice versa*, which mechanism obviates the above difficulties. The chief difference of our mechanism from the Löwdin mechanism is that, in the process of mutagenic tautomerization through sequential proton transfer, the DNA bases shift laterally relative each other into the DNA minor or major grooves, leading to the wobble configuration which contains the mutagenic tautomers [19]. Moreover, it turned out that a similar mechanism works also for the mutagenic tautomerization of purine-purine [21], pyrimidine-pyrimidine [22, 23] and purine-pyrimidine [24–27] DNA base mispairs, which are active players in the field of spontaneous point mutagenesis.

This allows us to assume that it is the intrapair tautomeric transition of the wobble pairs from the main tautomeric form into the rare one with a WC configuration or close to it, and *vice versa*, which is the key to understanding the microstructural mechanisms for spontaneous transitions and transversions during DNA biosynthesis [19–27]. Theoretical analyses of such mechanisms have been experimentally confirmed in part for the A∙C(w) and G∙T(w) purine-pyrimidine pairs [28–31].

This paper uses QM/QTAIM methods to explore new pathways for mutagenic tautomerization of the classical Watson-Crick A·Т(WC), reverse Watson-Crick A·Т(rWC), Hoogsteen A·Т(H) and reverse Hoogsteen A·Т(rH) base pairs with a remarkable biological meaning (for more details, see Refs. [32–49]). These are controlled by transition states with a quasi-orthogonal structure (symmetry C_1_) which are highly polar tight ion pairs (A^-^, N6H_2_–deprotonated)∙(T^+^, O4/O2-deprotonated).

## Computational methods

The geometries of all the investigated DNA base pairs and transition states (TSs) were optimized using the Gaussian’09 package [50]. The B3LYP/6-311++G(d,p) level of theory [51–55] was used. This level of theory has successfully proved itself for calculations of similar systems [56–63]. The study included harmonic frequency calculations (using a scaling factor of 0.9668 [64–66]) and intrinsic reaction coordinate (IRC) analysis in the forward and reverse directions from each ТS using a Hessian-based predictor-corrector integration algorithm [67] at the B3LYP/6-311++G(d,p) level of theory successfully applied in the previous studies [16, 17, 68, 69]. Local minima and TSs (localized by the synchronous transit-guided quasi-Newton method [70]) were confirmed as such by the absence or presence, respectively, of one imaginary frequency. Standard TS theory was applied to estimate the activation barriers for the tautomerisation reactions [71]. Single point electronic energy calculations were performed for the B3LYP geometries at the MP2/aug-cc-pVDZ level of theory [72, 73]. MP2 has been successfully applied to gain chemical information about similar proton transfer reactions in DNA systems [74–79]. The choice of the MP2 level of theory is caused by the insignificant errors in comparison with CCSD(T) method, that was convincingly shown in the benchmark works of Hobza and Šponer [80, 81].

We have performed investigations for the isolated H-bonded pairs of nucleotide bases, that adequately reflects the processes occurring in real duplex environment [14, 30, 31]. At this we relied on experience received in the previous works [82–85] devoted to related topics and systems, where the negligibly small impact of the stacking and sugar-phosphate backbone on the tautomerisation processes has been shown.

The Gibbs free energy G for all structures was obtained in the following way:

G=Eel+Ecorr (1)

where E_el_ = electronic energy, while E_corr_ = thermal correction to Gibbs free energy.

Electronic interaction energies ΔE_int_ were calculated at the MP2/6-311++G(2df,pd) level of theory as the difference between the total energy of the base pair and energies of the monomers and corrected for the basis set superposition error (BSSE) [86,87] through the counterpoise procedure [88,89] without consideration of the deformation energies of the monomers due to their relatively small values [90].

Bader’s quantum theory of Atoms in Molecules (QTAIM) [91–96] was applied to analyse the electron density distribution, using the AIMAll package [97] for the wave functions obtained at the B3LYP/6-311++G(d,p) level of theory. Presence of a bond critical point (BCP), namely, the so-called (3,-1) BCP, and a bond path between non-covalently connected atoms, as well as a positive value of the Laplacian at this BCP (Δρ>0), were considered as criteria for formation of an H-bond or attractive van der Waals contact [98–100].

The energies of the attractive van der Waals contacts [101, 102] in TSs for tautomeric transitions of the base pairs were calculated by the empirical Espinosa-Molins-Lecomte (EML) formula [103, 104] based on the electron density distribution at the (3,-1) BCPs of the specific contacts:

E=0.5V(r) (2)

where V(r) = value of a local potential energy at the (3,-1) BCP.

Energies of conventional AH···B H-bonds were evaluated by the empirical Iogansen formula [105]:

$$E_{AH\cdot \cdot \cdot B}=0.33\cdot \sqrt{\Delta \nu -40},$$ (3)

where Δν = magnitude of the stretching frequency shift for the AH H-bonded group involved in the AH···B H-bond relative to the unbound group. Partial deuteration was applied in order to avoid the effect of vibrational resonances [106–114].

The atom numbering scheme for the DNA bases is as per convention [108].

## Results and discussion

**Fig 1:**
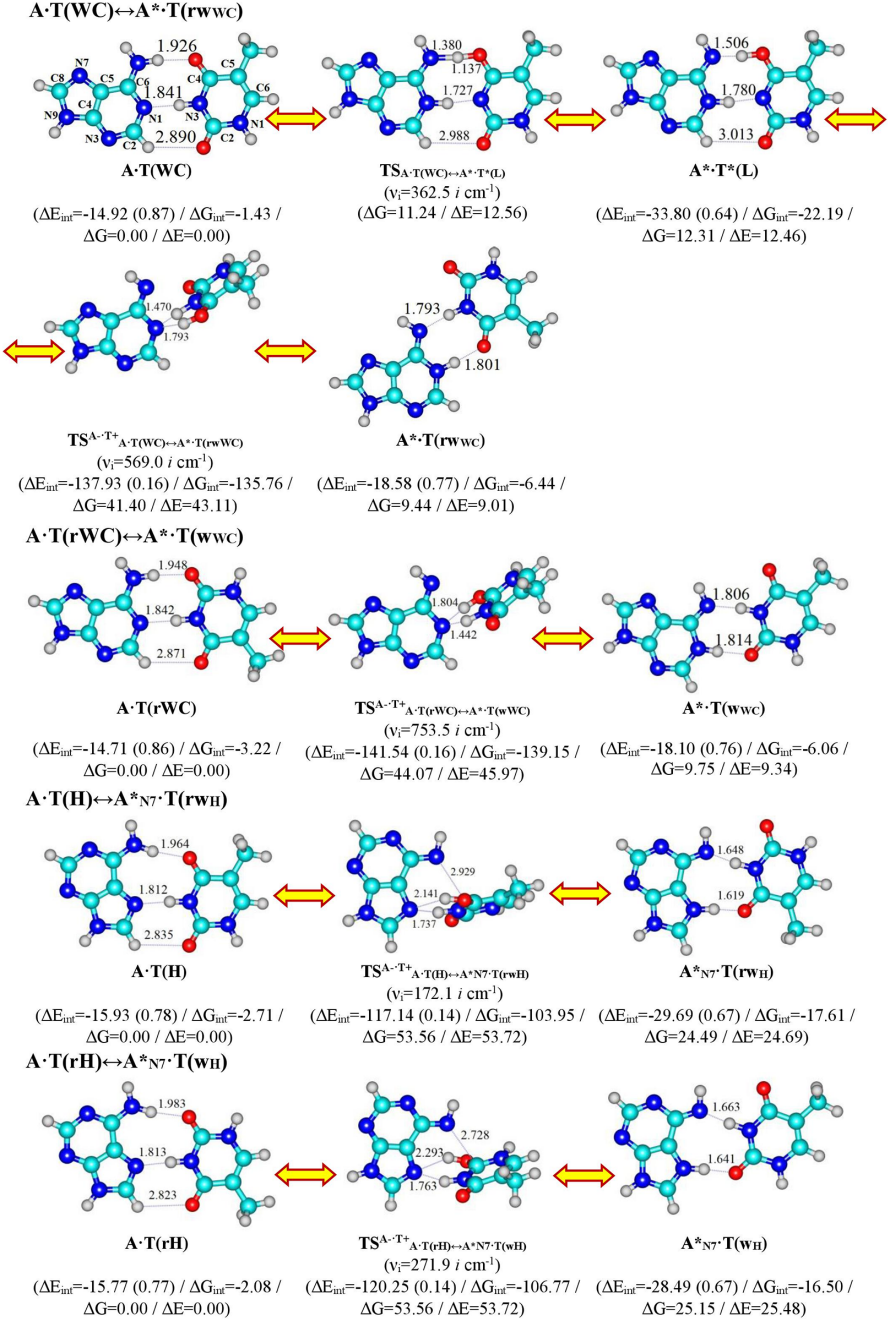
Geometrical structures of the stationary points on the discovered pathways of the tautomerizations *via* the sequential proton transfer in the four biologically important A·Т DNA base pairs through the TSs with quasi-orthogonal oriented bases. Electronic ΔE_int_ (contribution of the total energy of the intermolecular specific contacts) and Gibbs free ΔG_int_ energies of the interaction (MP2/6-311++G(2df,pd)//B3LYP/6-311++G(d,p) level of theory, in kcal∙mol^-1^), relative Gibbs free energies ΔG and electronic energies ΔE (in kcal∙mol^-1^), imaginary frequencies *ν*_*i*_ at the TSs of the conformational transitions (MP2/aug-cc-pVDZ//B3LYP/6-311++G(d,p) level of theory in the continuum with ε = 1 at T = 298.15 К) are presented below complexes in brackets. Dotted lines indicate AH···B H-bonds and attractive A···B van der Waals contacts—their lengths H···B and A···B are presented in angstroms (for their more detailed physico-chemical characteristics see Table 2); carbon atoms are in light-blue, nitrogen—in dark-blue, hydrogen—in grey and oxygen—in red.

**Fig 2:**
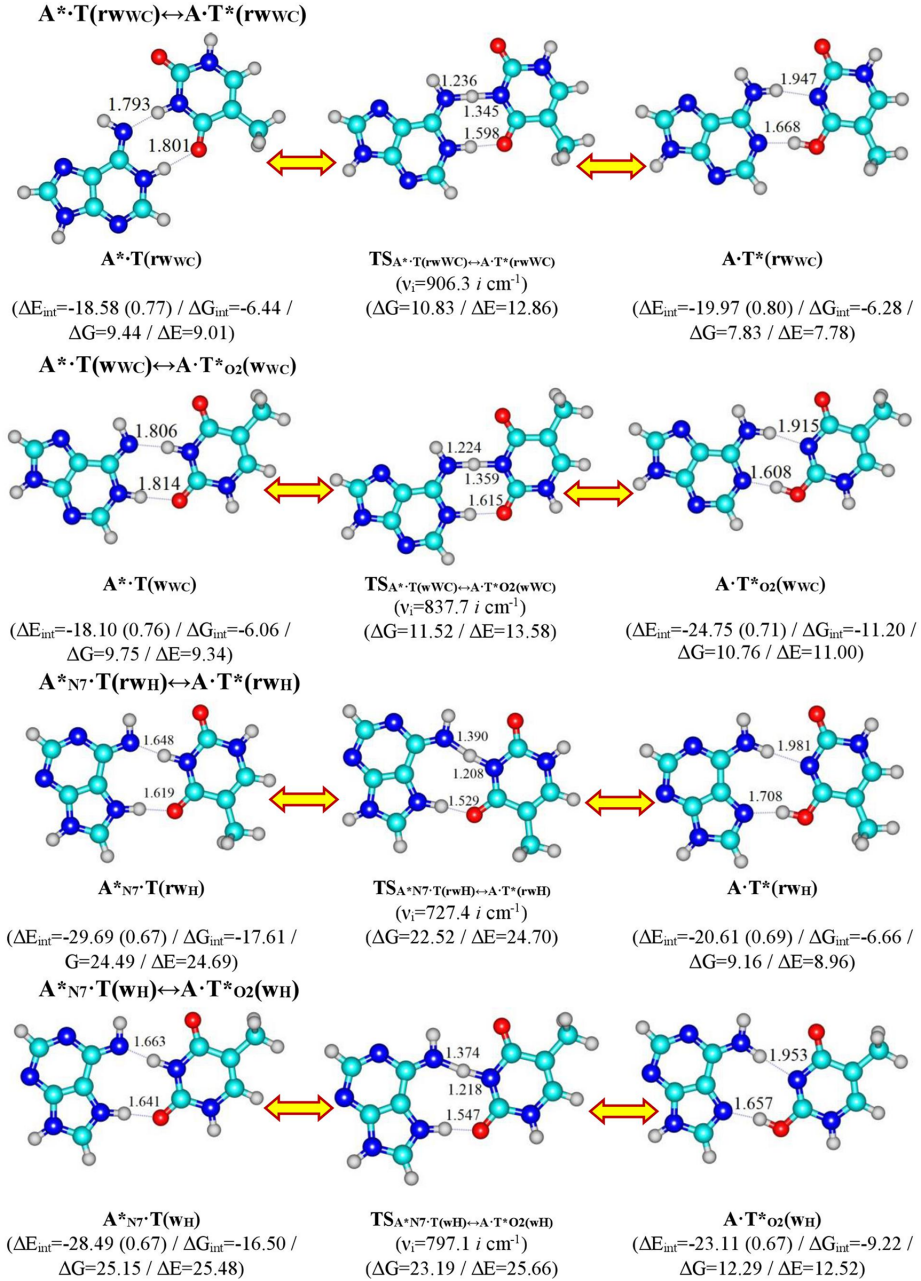
Geometrical structures of the stationary points on the pathways of the tautomerizations *via* the double proton transfer in the products of the discovered tautomerizations of the classical A∙T DNA base pairs. For the detailed designations see Fig 1.

These novel pathways for the mutagenic tautomerization of four biologically important A∙T DNA base pairs—Watson-Crick A·Т(WC), reverse Watson-Crick A·Т(rWC), Hoogsteen A·Т(H) and reverse Hoogsteen A·Т(rH) [32–49]–are portrayed in Figs 1 and 2, with data entered into Tables 1–3.

**Table 1:** Energetic characteristics (in kcal∙mol^-1^) of the discovered mutagenic tautomerizations of the biologically important A·Т DNA base pairs *via* the single and double proton transfers obtained at the MP2/aug-cc-pVDZ//B3LYP/6-311++G(d,p) level of QM theory in the continuum with ε = 1 under normal conditions (see Figs 1 and 2).

Tautomeric transition	ν_i_^a^	ΔG^b^	ΔE^c^	ΔΔG_TS_^d^	ΔΔE_TS_^e^	ΔΔG^f^	ΔΔE^g^
A∙T(WC)↔A*∙T*(L)	362.5	12.31	12.46	11.24	12.56	-1.07	0.10
A*∙T*(L)↔A*∙T(rw_**WC**_**)**	569.0	9.44	9.01	41.40	43.11	31.95	34.10
A·T(rWC)↔A*·T(w_**WC**_**)**	753.5	9.75	9.34	44.07	45.97	34.32	36.63
A·Т(H)↔A*_N7_**·Т(rw**_**H**_**)**	172.1	24.49	24.69	52.59	52.49	28.10	27.80
A·Т(rH)↔A*_N7_**·Т(w**_**H**_**)**	271.9	25.15	25.48	53.56	53.72	28.41	28.24
A*∙T(rw_**WC**_)↔A∙T*(rw_**WC**_**)**	906.3	-1.61	-1.24	1.39	3.85	3.00	5.08
A*·T(w_**WC**_)↔A·T*_O2_**(w**_**WC**_**)**	837.7	1.01	1.66	1.77	4.24	0.76	2.58
A*_N7_**·Т(rw**_**H**_)↔A·Т*(rw_**H**_**)**	727.4	-15.34	-15.73	-1.98	0.01	13.36	15.73
A*_N7_**·Т(w**_**H**_)↔A·Т*_O2_**(w**_**H**_**)**	797.1	-12.87	-12.96	-1.97	0.18	10.90	13.14

^a^Imaginary frequency at the TS of the tautomeric transition, cm^-1^.

^b^The Gibbs free energy of the product relatively the reactant of the tautomeric transition (T = 298.15 K).

^c^The electronic energy of the product relatively the reactant of the tautomeric transition.

^d^The Gibbs free energy barrier for the forward tautomeric transition.

^e^The electronic energy barrier for the forward tautomeric transition.

^f^The Gibbs free energy barrier for the reverse tautomeric transition.

^g^The electronic energy barrier for the reverse tautomeric transition.

**Table 2:** Electron-topological, geometrical and energetic characteristics of the intermolecular specific contacts—H-bonds and attractive van der Waals (vdW) contacts in the investigated DNA base pairs and TSs of their tautomeric transformations obtained at the B3LYP/6-311++G(d,p) level of QM theory (ε = 1) (see Figs 1 and 2).

Complex	AH···B H-bond / A···B vdW contact	*ρ*^a^	*Δρ*^b^	*100∙ε*^c^	*d*_*A⋯B*_^d^	*d*_*H⋯B*_^e^	∠AH⋯B^f^	*E*_*AH···B*_ *E*_*A···B*_^g^	μ^h^
**A·Т(WC)** [16, 19]	N6H⋯O4	0.026	0.093	4.39	2.946	1.926	173.5	4.65	1.88
	N3H⋯N1	0.040	0.093	6.49	2.886	1.841	178.8	7.58	
	C2H⋯O2	0.004	0.014	3.40	3.975	2.890	132.3	0.74*	
**TS**_A·T(WC)↔A*·T*(L)_	N1H⋯N3	0.052	0.101	6.08	2.775	1.727	171.4	9.62**	0.38
	C2H⋯O2	0.004	0.012	14.48	3.722	2.988	125.3	0.61*	
A*∙T*(L)	O4H⋯N6	0.087	0.065	4.56	2.578	1.506	174.8	13.47	0.78
	N1H⋯N3	0.045	0.101	6.24	2.825	1.780	171.1	7.73	
	C2H⋯O2	0.003	0.012	3.40	4.098	3.013	125.1	0.57*	
**TS**^**A-·T+**^_A·T(WC)↔A*·T(rwWC)_	N3^+^H⋯N1^-^	0.100	0.026	5.89	2.583	1.470	158.1	13.06	7.38
	O4^+^H⋯N1^-^	0.045	0.092	10.15	2.740	1.793	154.7	8.85	
A*∙T(rw_**WC**_**)**	N3H⋯N6	0.044	0.095	6.22	2.844	1.793	174.7	8.53	3.23
	N1H⋯O4	0.035	0.117	3.55	2.832	1.801	177.3	5.82	
**TS**_A*·T(rwWC)↔A·T*(rwWC)_	N1H⋯O4	0.061	0.142	3.32	2.663	1.598	179.3	11.61**	3.78
A∙T*(rw_**WC**_**)**	N6H⋯N3	0.030	0.087	7.07	2.682	1.668	170.4	5.76	2.52
	O4H⋯N1	0.059	0.096	5.10	2.955	1.947	167.0	10.21	
**A·Т(rWC)** [42]	N6H⋯O2	0.024	0.088	5.26	2.962	1.949	172.9	4.38	2.40
	N3H⋯N1	0.039	0.093	6.51	2.887	1.843	177.7	7.55	
	C2H⋯O4	0.004	0.014	3.32	3.696	2.872	132.8	0.77*	
**TS**^**A-·T+**^_A·T(rWC)↔A*·T(wWC)_	N3^+^H⋯N1^-^	0.107	0.005	5.60	2.574	1.442	158.2	13.24	6.83
	O2^+^H⋯N1^-^	0.043	0.090	9.82	2.741	1.804	152.8	8.82	
A*·T(w_**WC**_**)**	N3H⋯N6	0.042	0.095	6.21	2.858	1.806	173.3	8.31	4.29
	N1H⋯O2	0.034	0.115	4.40	2.845	1.814	177.0	5.49	
**TS**_A*·T(wWC)↔A·T*O2(wWC)_	N1H⋯O2	0.058	0.141	4.10	2.676	1.615	179.2	10.94***	5.33
A·T*_O2_**(w**_**WC**_**)**	N6H⋯N3	0.034	0.088	1.71	2.944	1.915	167.1	6.19	3.96
	O2H⋯N1	0.071	0.081	0.86	2.644	1.608	171.9	11.43	
**A·Т(H)** [42]	N6H´⋯O4	0.023	0.086	3.93	2.972	1.963	170.6	4.18	6.16
	N3H⋯N7	0.041	0.099	5.75	2.853	1.811	175.9	7.39	
	C8H⋯O2	0.005	0.016	7.71	3.524	2.835	121.7	0.83*	
**TS**^**A-·T+**^_A·Т(H)↔A*N7·T(rwH)_	N3^+^H⋯N7^-^	0.050	0.097	4.51	2.754	1.737	158.7	8.98	12.65
	O4^+^H⋯N7^-^	0.019	0.058	9.97	3.021	2.141	147.7	5.18	
	O4^+^⋯N6^-^	0.013	0.043	68.81	2.929	-	-	2.58*	
A*_N7_**·Т(rw**_**H**_**)**	N3H⋯N6	0.062	0.090	5.55	2.731	1.648	174.5	11.26	9.42
	N7H⋯O4	0.055	0.147	2.33	2.671	1.619	175.8	8.61	
**TS**_A*N7·Т(rwH)↔A·Т*(rwH)_	N7H⋯O4	0.070	0.151	2.34	2.603	1.529	175.7	13.76**	8.37
A·Т*(rw_**H**_**)**	N6H´⋯N3	0.027	0.082	7.62	3.000	1.981	175.7	5.09	7.36
	O4H⋯N7	0.052	0.102	4.48	2.702	1.708	166.4	9.18	
**A·Т(rH)** [42]	N6H´⋯O2	0.022	0.082	4.95	2.994	1.986	170.9	3.90	5.67
	N3H⋯N7	0.041	0.099	5.80	2.856	1.815	176.9	7.34	
	C8H⋯O4	0.005	0.017	7.97	3.517	2.825	121.9	0.86*	
**TS**_A·Т(rH)↔A*N7·Т(wH)_	N3^+^H⋯N7^-^	0.047	0.098	3.26	2.757	1.763	154.8	8.46	10.36
	O2^+^H⋯N7^-^	0.014	0.044	12.37	3.103	2.293	139.2	4.38	
	O2^+^⋯N6^-^	0.018	0.055	78.70	2.829	-	-	3.71*	
A*_N7_**·Т(w**_**H**_**)**	N3H⋯N6	0.060	0.092	5.58	2.743	1.663	175.7	10.97	10.35
	N7H⋯O2	0.051	0.145	3.17	2.689	1.641	176.3	8.09	
**TS**_A*N7·Т(wH)↔A·Т*O2(wH)_	N7H⋯O4	0.067	0.152	3.15	2.615	1.547	176.3	12.95**	9.46
A·Т*_O2_**(w**_**H**_**)**	N6H´⋯N3	0.029	0.086	7.38	2.974	1.953	176.4	5.38	8.23
	O2H⋯N7	0.059	0.100	4.48	2.664	1.657	168.0	10.16	

^a^The electron density at the (3,-1) BCP of the specific contact, a.u.

^b^The Laplacian of the electron density at the (3,-1) BCP of the specific contact, a.u.

^c^The ellipticity at the (3,-1) BCP of the specific contact.

^d^The distance between the A and B atoms of the of the AH···B / A···B specific contact, Å.

^e^The distance between the H and B atoms of the AH···B H-bond, Å.

^f^The H-bond angle, degree.

^g^Energy of the specific contact, calculated by Iogansen’s [105], Espinose-Molins-Lecomte [103, 104] (marked with an asterisk) or Nikolaienko-Bulavin-Hovorun [109] (marked with double asterisk) formulas, kcal∙mol^-1^.

^h^The dipole moment of the complex, D.

**Table 3:** Selected geometrical parameters, characterizing the non-planarity of the discovered mutagenic tautomerizations of the biologically important A·Т DNA base pairs *via* the single and double proton transfers, obtained at the B3LYP/6-311++G(d,p) level of QM theory in the continuum with ε = 1.

TS of tautomerisation	Dihedral angles, degree	
	(A)N7C5(T)N3C4	(T)HO4/O2C4/C2N3
TS^A-·T+^_A·T(WC)↔A*·T(rwWC)_	85.0	-9.3
TS^A-·T+^_A·T(rWC)↔A*·T(wWC)_	60.4	11.8
TS^A-·T+^_A·Т(H)↔A*N7·T(rwH)_	-99.0	-19.9
TS^A-·T+^_A·Т(rH)↔A*N7·T(wH)_	-119.4	40.1

Conformers of the A∙T base pairs remain plane symmetric structures along the entire IRC of tautomerization. This also holds for base pairs tautomerising *via* proton transfer along intermolecular H-bonds as per currently known mechanisms for mutagenic tautomerization of WC pairs [16, 17, 19, 49].

The A∙T(WC) / A∙T(rWC) / A∙T(H) / A∙T(rH) ↔ A*∙Т(rw_WC_) / A*∙Т(w_WC_) / A*_N7_∙Т(rw_H_) / A*_N7_∙Т(w_H_) tautomerisation reactions occur *via* the initial migration of proton localized at the N6 atom of the N6H_2_ amino group, leading to the formation of the A^+^∙Т^-^ ion pair and significant change of the mutual orientation of the bases within the pair, i.e. mutual transformation of the *cys / trans*↔*trans / cys*-orientation of the N1H and N9H bonds relative to each other (Fig 1). Our new mechanism is controlled by the TSs having quasi-orthogonal structures (symmetry C_1_). Further proton transfers to the N1/N7 nitrogen atom causing the rotation of the base and formation of the terminal wobble base mispair. Each of these tautomeric conversions is followed by the asynchronous DPT along the intermolecular H-bonds in the wobble base mispairs (Fig 2).

In all four cases of the novel A∙T(WC) / A∙T(rWC) / A∙T(H) / A∙T(rH) ↔ A*∙Т(rw_WC_) / A*∙Т(w_WC_) / A*_N7_∙Т(rw_H_) / A*_N7_∙Т(w_H_) tautomerisation reactions, the TSs are highly polar (~ 6.8–12.7 D) tight ion pairs (energy of interaction between bases in the pairs ~117–142 kcal∙mol^-1^) (Table 2). These TSs are (A^-^, N6H_2_-deprotonated)∙(T^+^, O4/O2-protonated) ion pairs. In the TS^A-·T+^_A·T(WC)↔A*·T(rwWC)_ / TS^A-·T+^_A·T(rWC)↔A*·T(wWC)_ transition states of tautomerisation the (T)N3^+^H⋯N1^-^(A) (13.06 / 13.24) and (T)O4^+^ / O2^+^H⋯N1^-^(A) (8.85 / 8.82 kcal∙mol^-1^) are observed, while for the TS^A-·T+^_A·Т(H)↔A*N7·T(rwH)_ / TS^A-·T+^_A·Т(rH)↔A*N7·T(wH)_ transition states, the (T)N3^+^H⋯N7^-^(A) (8.98 / 8.46) and (T)O4^+^ / O2^+^H⋯N7^-^(A) (5.18 / 4.38) H-bonds are supplemented by attractive (T)O4^+^/O2^+^⋯N6^-^(A) (2.58 / 3.71 kcal∙mol^-1^) van der Waals contacts (Fig 1, Table 2). At this, the (T)N3^+^H⋯N1^-^/N7^-^(A) H-bonds (~ 8.5–13.0 kcal∙mol^-1^) are significantly stronger than other specific contacts with increased ellipticity. The weakest among them are the attractive (T)O4^+^/O2^+^⋯N6^-^(A) (2.58 / 3.71 kcal∙mol^-1^) van der Waals contacts (Table 2).

All TS_A*·T(rwWC)↔A·T*(rwWC)_, TS_A*·T(wWC)↔A·T*O2(wWC)_, TS_A*N7·Т(rwH)↔A·Т*(rwH)_ and TS_A*N7·Т(wH)↔A·Т*O2(wH)_ of the DPT reactions are stabilized by the N6-H-N3 covalent bridge and one-single intermolecular H-bond—N1H⋯O4 (11.61), N1H⋯O2 (10.94), N7H⋯O4 (13.76) and N7H⋯O4 (12.95 kcal∙mol^-1^), accordingly (Fig 2, Table 2).

The non-canonical CH⋯O H-bonds [110, 111] have been registered in the initial complexes of the tautomerisation: A·Т(WC)–C2H⋯O2 (0.74), A*·Т*(L)–C2H⋯O2 (0.57), A·Т(rWC)–C2H⋯O4 (0.77), A·Т(H)–C8H⋯O2 (0.83), A·Т(rH)–C8H⋯O4 (0.86 kcal∙mol^-1^), which are characterized by low energies E_CH···O_, estimated by the Espinose-Molins-Lecomte formula [103, 104], decreased electron-topological parameters (ρ, Δρ, 100∙ε) and angles (∠AH⋯B), but increased intermolecular distances (d_C⋯O_ and d_H⋯O_) in comparison with the canonical H-bonds (for more details see Table 2).

In general, the values of the electron density ρ at the (3,-1) BCPs of the intermolecular H-bonds range from 0.013 a.u. up to the 0.107 a.u.; the values of the Laplacian of the electron density Δρ at the (3,-1) BCPs are positive for all intrapair H-bonds and lie within a wide range from 0.005 a.u. up to the 0.152 a.u., demonstrating that H-bonds are attractive closed-shell ineractions; the value of the ellipticity ε varies in the range 0.79–8.6·10^−3^ (Table 2).

The classical geometrical criteria are satisfied for all canonical H-bonds in the investigated base mispairs and TSs of their interconversions: d_A···B_ (2.574–3.103 Å), d_H···B_ (1.442–2.293 Å) and ∠AH⋯B (139.2–179.3°) (Table 2).

Interestingly, the energy of the intermolecular specific contacts (H-bonds and attractive van der Waals contacts) constitute only a minor part of the electronic energy of monomeric interactions for all these H-bonded structures (~14–0.87%) (see Figs 1 and 2). This agrees with previous results for other H-bonded base pairs [112].

All tautomeric transitions in this work are dipole-active, being accompanied by significant changes in dipole moment of the tautomerizing structures along the IRC (0.38–12.65 D), achieving maximum values for each tautomeric transition at its TS (7.38, 6.83, 12.65 and 10.636 D, accordingly) (Table 2). The Gibbs free energy of activation for the A∙T(WC)/A∙T(rWC)↔A*∙Т(rw_WC_)/A*∙Т(w_WC_) tautomerisations (~ 43.5 kcal∙mol^-1^) is noticeably lower than for the A∙T(H)/A∙T(rH)↔A∙T*(rw_H_)/A∙T*_O2_(w_H_) tautomerisations (~ 53.0 kcal∙mol^-1^) (Figs 1 and 2).

Note that only one case of mutagenic tautomerization, the A∙T(WC)↔A*∙Т(rw_WC_) reaction, occurs by participation of the dynamically unstable intermediate A*∙Т*(L) (a Löwdin’s base pair [3, 4]). The other three A∙T DNA base pairs—A∙T(rWC), A∙T(H) and A∙T(rH)–do not tautomerise *via* the Löwdin’s mechanism. For these three pairs, the local minima corresponding to the tautomerized A*∙Т*_О2_, A*_N7_∙Т* and A*_N7_∙Т*_O2_ base pairs are absent on the energy hypersurface. This observation is independent of the level of QM theory used.

It should be noted that three out of four tautomerization processes of the A∙T base pairs do not complete with formation of the A*∙Т(rw_WC_), A*_N7_∙Т(rw_H_) and A*_N7_∙Т(w_H_) mispairs (Fig 2 and Table 1). These plane-symmetric wobble pairs (symmetry C_s_) tautomerise further *via* the DPT mechanism along neighboring intermolecular H-bonds into the energetically-favorable plane-symmetric A∙T*(rw_WC_), A∙T*(rw_H_) and A∙T*_O2_(w_H_) DNA base mispairs, respectively (Fig 2, Tables 1 and 2). These processes occur *via* a concerted asynchronous mechanism with proton transfer along the intermolecular (T)N3H⋯N6(A) H-bonds, which, in fact, is a rate-limiting stage. It is noteworthy that the A*_N7_·Т(rw_H_)→A·Т*(rw_H_) and A*_N7_·Т(w_H_)→A·Т*_O2_(w_H_) tautomerisations are barrier-less (ΔΔG_TS_ = -1.98 and -1.97 kcal∙mol^-1^) (Table 1), while the activation barriers for the A*∙T(rw_WC_)↔A∙T*(rw_WC_) (1.39) and A*·T(w_WC_)↔A·T*_O2_(w_WC_) (1.77) are significantly lower than for the novel tautomerisation reactions (41.40–53.56 kcal∙mol^-1^), but are comparable with the values for the other DPT reactions [113]: from 2.42 for A*∙G*_syn_↔A∙G*_syn_ [100] to 10.29 kcal∙mol^-1^ for A∙T↔A*∙T* [16] DPT tautomerisations.

It is thus possible to say that the tautomerization processes described here terminate with the mutagenic tautomerization of both T and A DNA bases with further formation of the classical mutagenic tautomers Т*, Т*_О2_ [16, 19, 27, 64, 102, 106] and A* [16, 19–22, 25, 26, 100, 114], respectively. In this case, the A*_N7_·Т(rw_H_)↔A·Т*(rw_H_) and A*_N7_·Т(w_H_)↔A·Т*_O2_(w_H_) tautomeric equilibria are completely shifted to the right. For the two other cases, the following proportions are observed: A*·Т(rw_WC_) (6.9%) ↔ A·Т*(rw_WC_) (93.1%) and A*·Т(w_WC_) (83.6%) ↔ A·Т*_O2_(w_WC_) (16.4%).

Gibbs free energies (in kcal∙mol^-1^) and populations of the investigated base mispairs yield the order: A·Т(H) (0.00) < A·Т(rH) (0.22/0.63) < A·Т(WC) (1.05/0.21) < A·Т(rWC) (1.31/0.16) < A∙T*(rw_WC_) (8.83/3.77∙10^−7^) < A·Т*(rw_H_) (8.96/1.92∙10^−7^) < A*∙T(rw_WC_) (10.07/2.47∙10^−8^) < A*·T(w_WC_) (10.65/1.13∙10^−8^) < A·T*_O2_(w_WC_) (12.31/2.07∙10^−9^) < A·Т*_O2_(w_H_) (12.74/6.13∙10^−10^) < A*∙T*(L) (13.51/1.95∙10^−10^) < A*_N7_·Т(rw_H_) (24.69/1.08∙10^−18^) < A*_N7_·Т(w_H_) (25.70/2.24∙10^−19^). Notably, populations of the wobble A∙T*(rw_WC_), A·Т*(rw_H_), A*∙T(rw_WC_), A*·T(w_WC_), A·T*_O2_(w_WC_), A·Т*_O2_(w_H_) (12.74/6.13∙10^−10^) and A*∙T*(L) tautomerised states, fitting into the range of the frequencies of the spontaneous point mutations observed experimentally (10^−11^–10^−9^) [115–117], point on their involvement into the processes of the origin of the spontaneous point mutations.

Notably, the methyl group of the T DNA base does not change its orientation during all these tautomerisation processes without exception. Moreover, the heterocycles of the DNA bases remain planar, despite their ability for out-of-plane bending [118–120].

A relatively small non-planarity of the pyrimidine ring of the protonated T^+^ base occurs only in the TS^A-·T+^_A·T(WC)↔A*·T(rwWC)_, TS^A-·T+^_A·T(rWC)↔A*·T(wWC)_, TS^A-·T+^_A·Т(H)↔A*N7·T(rwH)_ and TS^A-·T+^_A·Т(rH)↔A*N7·T(wH)_ transition states. The maximum value of the non-planar dihedral angle reaches 2.5° (C2-N3-C4-C5), 3.1° (N1-C2-N3-C4), 3.7° (C2-N3-C4-C5) and 7.8° (N1-C2-N3-C4), respectively. Another structural feature of the protonated T^+^ base in these TSs is the deviation of the О4^+^H / О2^+^H hydroxyl group from the plane of the pyrimidine ring (the dihedral angles range from 9.3 to 40.1°) (Table 3).

## Conclusions and perspectives

Novel pathways for mutagenic tautomerization of four classical A∙T DNA base pairs, followed by the significant changes of base orientation within the pair, have been predicted by these QM results. The transition states with quasi-orthogonal structure (symmetry C_1_) are highly polar tight ion pairs (A^-^, N6H_2_-deprotonated)∙(T^+^, O4/O2-protonated). The tautomerization products—the A*∙Т(rw_WC_), A*_N7_∙Т(rw_H_) and A*_N7_∙Т(w_H_) pairs—further transform *via* concerted asynchronous double proton transfer into the energetically favorable wobble A∙T*(rw_WC_), A∙T*(rw_H_) and A∙T*_O2_(w_H_) mispairs (symmetry C_s_), respectively. Moreover, it was established in our recent papers, that wobble A*∙T(rw_WC_) base mispair can also be formed from the reverse A∙T(rWC) base pair [20], A*·T(wWC) base mispair—from the canonical A∙T(rWC) base pair [19] and A*_N7_·Т(w_H_) base mispair—from the Hoogsteen A∙T(H) base pair [20].

We are currently engaged in elaborating this topic in order to discover biologically important H-bonded nucleobase pairs, for which the mechanism of mutagenic tautomerization plays a key role. Moreover, we suggested that novel mechanism of mutagenic tautomerization presented in this study could lead to the conversion of an anti-parallel DNA helix to a parallel DNA helix. We also consider investigation of these tautomerisation mechanism by the participation of the modified A∙T base pairs [121–124] as a task for the future.

## Supporting information

S1 Dataset Cartesian coordinates of the investigated complexes: A·Т(WC); TS_A·T(WC)↔A*·T*(L)_; A*∙T*(L); TSA-·T+A·T(WC)↔A*·T(rwWC); A*∙T(rw_WC_); TS_A*·T(rwWC)↔A·T*(rwWC)_; A∙T*(rw_WC_); A·Т(rWC); TSA-·T+A·T(rWC)↔A*·T(wWC); A*·T(w_WC_); TS_A*·T(wWC)↔A·T*O2(wWC)_; A·T*_O2_(w_WC_); A·Т(H); TSA-·T+A·Т(H)↔A*N7·T(rwH); A*_N7_·Т(rw_H_); TS_A*N7·Т(rwH)↔A·Т*(rwH)_; A·Т*(rw_H_); A·Т(rH); TS_A·Т(rH)↔A*N7·Т(wH)_; A*_N7_·Т(w_H_); TS_A*N7·Т(wH)↔A·Т*O2(wH)_; A·Т*_O2_(w_H_). (DOC)
