# Supplementary material for: Novel pathway for mutagenic tautomerization of classical А∙Т DNA base pairs via sequential proton transfer through quasi-orthogonal transition states: A QM/QTAIM investigation
Source: PLoS One. 2018 Jun 27;13(6):e0199044. doi: 10.1371/journal.pone.0199044 (PMC6021055; doi:10.1371/journal.pone.0199044)
Supplement: S1 Dataset — (DOC) [file pone.0199044.s001.doc]

**SUPPORTING INFORMATION**

**Novel pathway for mutagenic tautomerization of classical А∙Т DNA base pairs *via* sequential proton transfer: A QM/QTAIM investigation**

Ol’ha O. Brovarets’^1,2^, Kostiantyn S. Tsiupa^1^ & Dmytro M. Hovorun^1,2*^

^a^Department of Molecular and Quantum Biophysics, Institute of Molecular Biology and Genetics, National Academy of Sciences of Ukraine, 150 Akademika Zabolotnoho Str., 03680 Kyiv, Ukraine
^b^Department of Molecular Biotechnology and Bioinformatics, Institute of High Technologies, Taras Shevchenko National University of Kyiv, 2-h Akademika Hlushkova Ave., 03022 Kyiv, Ukraine

*Corresponding author

E-mail: dhovorun@imbg.org.ua

Cartesian coordinates of the investigated complexes:

**А·Т(WC)**

7 -5.000340000 0.543270000 0.000007000

6 -5.244709000 -0.815249000 0.000012000

1 -6.250299000 -1.209023000 0.000018000

7 -4.151775000 -1.533591000 0.000009000

6 -3.130209000 -0.600803000 0.000001000

6 -1.724133000 -0.732931000 -0.000005000

7 -1.101936000 -1.922615000 -0.000005000

1 -1.656524000 -2.762561000 0.000003000

1 -0.083141000 -1.977062000 -0.000008000

7 -0.992864000 0.403488000 -0.000011000

6 -1.616933000 1.595949000 -0.000012000

1 -0.957957000 2.458979000 -0.000017000

7 -2.924919000 1.836262000 -0.000006000

6 -3.633095000 0.700375000 0.000000000

1 -5.679682000 1.288670000 0.000008000

7 3.899625000 1.567746000 0.000008000

6 4.593910000 0.379648000 0.000009000

1 5.673208000 0.475083000 0.000015000

6 3.975609000 -0.821142000 0.000003000

6 4.701510000 -2.133881000 0.000004000

1 5.783131000 -1.983600000 0.000007000

1 4.432128000 -2.728627000 0.876988000

1 4.432133000 -2.728626000 -0.876981000

6 2.511535000 -0.830417000 -0.000005000

8 1.840059000 -1.861467000 -0.000012000

7 1.892494000 0.414462000 -0.000005000

1 0.847540000 0.424332000 -0.000008000

6 2.510640000 1.647931000 0.000002000

8 1.918646000 2.710116000 0.000003000

1 4.383083000 2.453208000 0.000014000

**TS_A·T(WC)↔A*·T*(L)_**

7 -4.946721000 0.364458000 -0.000191000

6 -5.079847000 -1.008349000 -0.000164000

7 -3.926504000 -1.628125000 -0.000112000

6 -2.991080000 -0.617267000 -0.000105000

6 -1.562317000 -0.673727000 -0.000056000

7 -0.786405000 -1.719827000 -0.000005000

7 -0.997804000 0.594272000 -0.000066000

6 -1.701463000 1.754060000 -0.000115000

7 -3.006713000 1.849608000 -0.000161000

6 -3.601160000 0.632124000 -0.000153000

1 -5.683622000 1.053683000 -0.000230000

1 -6.047969000 -1.485938000 -0.000185000

1 -1.310645000 -2.588242000 -0.000003000

1 -1.091962000 2.651194000 -0.000115000

7 3.844504000 1.526986000 0.000127000

6 4.513936000 0.335398000 0.000185000

6 3.850246000 -0.846457000 0.000179000

6 4.529546000 -2.184963000 0.000242000

6 2.401983000 -0.749753000 0.000107000

8 1.725357000 -1.851038000 0.000100000

7 1.771176000 0.423946000 0.000048000

6 2.443636000 1.615009000 0.000056000

8 1.894815000 2.710269000 0.000005000

1 4.341593000 2.405635000 0.000132000

1 5.595927000 0.396821000 0.000236000

1 5.616008000 -2.070109000 0.000292000

1 4.243522000 -2.771609000 0.877481000

1 4.243608000 -2.771648000 -0.876999000

1 0.593907000 -1.737096000 0.000050000

1 0.056656000 0.627772000 -0.000029000

**A*∙T*(L)**

7 4.970460000 0.362011000 -0.000118000

6 5.104530000 -1.010435000 -0.000150000

1 6.072771000 -1.487724000 -0.000179000

7 3.950855000 -1.630162000 -0.000141000

6 3.014806000 -0.620089000 -0.000102000

6 1.582860000 -0.681771000 -0.000075000

7 0.802061000 -1.719270000 -0.000084000

1 1.335058000 -2.583663000 -0.000115000

7 1.022012000 0.593616000 -0.000034000

6 1.726199000 1.753250000 -0.000023000

1 1.117949000 2.651212000 0.000011000

7 3.030715000 1.848186000 -0.000047000

6 3.624392000 0.628529000 -0.000086000

1 5.706638000 1.051901000 -0.000118000

7 -3.854735000 1.535238000 0.000147000

6 -4.534612000 0.350937000 0.000152000

1 -5.615954000 0.421175000 0.000194000

6 -3.880342000 -0.837105000 0.000105000

6 -4.569551000 -2.170873000 0.000108000

1 -5.654902000 -2.047185000 0.000149000

1 -4.288700000 -2.759403000 -0.877422000

1 -4.288637000 -2.759432000 0.877598000

6 -2.435097000 -0.744574000 0.000050000

8 -1.769724000 -1.863584000 0.000007000

7 -1.789543000 0.413819000 0.000048000

6 -2.452448000 1.612493000 0.000098000

8 -1.895190000 2.701824000 0.000103000

1 -4.344261000 2.418378000 0.000189000

1 -0.704734000 -1.746832000 -0.000031000

1 -0.023103000 0.629423000 -0.000005000

**TS^A-·T+^_A·T(WC)↔A*·T(rwWC)_**

7 -4.784004000 0.138988000 -0.283062000

6 -4.886045000 -0.324458000 1.012048000

1 -5.841522000 -0.449091000 1.498895000

7 -3.717364000 -0.581676000 1.545925000

6 -2.803862000 -0.273140000 0.559332000

6 -1.361324000 -0.354138000 0.559915000

7 -0.556388000 -0.747359000 1.490139000

1 -1.093749000 -1.012817000 2.313310000

1 0.483619000 -1.173356000 -0.930818000

7 -0.793216000 0.058119000 -0.672714000

6 -1.568344000 0.489379000 -1.700541000

1 -1.021219000 0.800476000 -2.588544000

7 -2.877129000 0.575569000 -1.754293000

6 -3.443745000 0.176192000 -0.583676000

1 -5.533855000 0.408395000 -0.901005000

7 3.715009000 1.370933000 0.601789000

6 4.255491000 0.129322000 0.458216000

1 5.284069000 0.021626000 0.780704000

6 3.546315000 -0.913774000 -0.053349000

6 4.083414000 -2.306793000 -0.214231000

1 5.123242000 -2.358559000 0.113014000

1 3.499249000 -3.018715000 0.374064000

1 4.034295000 -2.631740000 -1.256593000

6 2.196754000 -0.601330000 -0.403605000

8 1.424076000 -1.537510000 -0.879142000

7 1.694287000 0.628884000 -0.272262000

1 0.549435000 0.618726000 -0.460380000

6 2.395512000 1.702172000 0.254563000

8 1.951787000 2.815214000 0.396833000

1 4.256182000 2.128624000 0.995127000

**A*∙T(rw_WC_)**

7 -5.161937000 0.130138000 -0.000152000

6 -5.072921000 -1.245468000 -0.000561000

1 -5.950926000 -1.873398000 -0.000822000

7 -3.833198000 -1.669194000 -0.000558000

6 -3.072546000 -0.522370000 -0.000153000

6 -1.641404000 -0.361602000 -0.000057000

7 -0.699927000 -1.241161000 -0.000366000

1 -1.083156000 -2.183059000 -0.000711000

1 1.069631000 -0.954291000 0.000381000

7 -1.298500000 1.002429000 0.000443000

6 -2.187399000 2.028305000 0.000656000

1 -1.745072000 3.018614000 0.000949000

7 -3.487262000 1.912247000 0.000530000

6 -3.875764000 0.608620000 0.000129000

1 -5.999516000 0.692370000 -0.000077000

7 4.325206000 -1.309308000 0.000116000

6 4.704786000 0.010519000 -0.000285000

1 5.774010000 0.186350000 -0.000595000

6 3.806857000 1.020118000 -0.000319000

6 4.184999000 2.471945000 -0.000811000

1 5.270320000 2.592894000 -0.000936000

1 3.778008000 2.981913000 -0.878158000

1 3.778137000 2.982456000 0.876276000

6 2.389307000 0.662473000 0.000131000

8 1.488034000 1.508270000 0.000075000

7 2.089669000 -0.689877000 0.000593000

1 -0.288799000 1.216964000 0.000399000

6 2.996533000 -1.732715000 0.000386000

8 2.693611000 -2.907961000 0.000462000

1 5.011157000 -2.049160000 0.000060000

**TS_A*·T(rwWC)↔A·T*(rwWC)_**

7 5.035079000 0.141930000 0.000121000

6 4.938309000 -1.234438000 0.000122000

1 5.813917000 -1.865851000 0.000147000

7 3.698881000 -1.655044000 0.000091000

6 2.944528000 -0.503528000 0.000068000

6 1.529442000 -0.321058000 0.000030000

7 0.599336000 -1.233363000 0.000008000

1 0.929157000 -2.190753000 0.000019000

1 -0.610554000 -0.978418000 -0.000020000

7 1.169009000 1.017489000 0.000017000

6 2.060399000 2.041021000 0.000038000

1 1.617638000 3.030570000 0.000023000

7 3.362739000 1.924265000 0.000072000

6 3.753072000 0.627668000 0.000086000

1 5.876663000 0.698774000 0.000141000

7 -4.148298000 -1.365212000 -0.000083000

6 -4.587928000 -0.067439000 -0.000096000

1 -5.663700000 0.066169000 -0.000116000

6 -3.725129000 0.973700000 -0.000084000

6 -4.155538000 2.410990000 -0.000098000

1 -3.767357000 2.936898000 -0.876872000

1 -5.245114000 2.493715000 -0.000115000

1 -3.767385000 2.936907000 0.876683000

6 -2.299178000 0.648659000 -0.000058000

8 -1.437643000 1.563837000 -0.000047000

7 -1.916659000 -0.659380000 -0.000046000

1 0.127651000 1.243917000 -0.000010000

6 -2.790653000 -1.713562000 -0.000057000

8 -2.452219000 -2.888908000 -0.000045000

1 -4.796577000 -2.138226000 -0.000092000

**A∙T*(rw_WC_)**

7 -5.099650000 0.315006000 -0.003032000

6 -5.115509000 -1.065632000 -0.001894000

1 -6.041655000 -1.620962000 -0.002884000

7 -3.918239000 -1.591680000 0.000382000

6 -3.066584000 -0.503074000 0.000905000

6 -1.655513000 -0.407972000 0.002965000

7 -0.847988000 -1.470984000 0.005846000

1 -1.249887000 -2.394355000 0.003717000

1 0.170252000 -1.345746000 0.003621000

7 -1.123938000 0.841254000 0.002444000

6 -1.940754000 1.911837000 0.000197000

1 -1.438857000 2.874750000 0.000008000

7 -3.266848000 1.934625000 -0.001691000

6 -3.778282000 0.695139000 -0.001292000

1 -5.893326000 0.937373000 -0.004834000

7 4.273306000 -1.295065000 -0.002109000

6 4.664934000 0.010328000 -0.001625000

1 5.733421000 0.191011000 -0.002331000

6 3.753621000 1.016225000 -0.000358000

6 4.116097000 2.473518000 0.000179000

1 5.200829000 2.602027000 -0.000529000

1 3.708480000 2.982807000 -0.877246000

1 3.709733000 2.981857000 0.878732000

6 2.375859000 0.582384000 0.000383000

8 1.466537000 1.534453000 0.001499000

7 1.999634000 -0.679947000 0.000049000

1 0.511467000 1.169194000 0.001472000

6 2.922893000 -1.697613000 -0.001172000

8 2.641390000 -2.884199000 -0.001463000

1 4.951619000 -2.043269000 -0.002962000

**А·Т(rWC)**

7 -4.900413000 -1.042939000 -0.000499000

6 -5.362768000 0.257599000 -0.000400000

1 -6.419099000 0.482237000 -0.000581000

7 -4.401521000 1.144487000 -0.000022000

6 -3.241676000 0.390522000 0.000110000

6 -1.876113000 0.749360000 0.000393000

7 -1.457266000 2.025711000 0.000733000

1 -2.141891000 2.763459000 0.000263000

1 -0.462783000 2.247384000 0.000319000

7 -0.969576000 -0.251977000 0.000364000

6 -1.390513000 -1.530539000 0.000036000

1 -0.598985000 -2.273942000 -0.000022000

7 -2.642040000 -1.980451000 -0.000275000

6 -3.525847000 -0.975336000 -0.000209000

1 -5.449403000 -1.888951000 -0.000917000

7 3.630805000 1.690538000 -0.000490000

6 4.544890000 0.655712000 -0.000623000

1 5.584426000 0.960721000 -0.001073000

6 4.169167000 -0.639410000 -0.000202000

6 5.132653000 -1.788466000 -0.000301000

1 6.166257000 -1.436169000 -0.000884000

1 4.979129000 -2.423303000 0.876467000

1 4.978320000 -2.423824000 -0.876549000

6 2.730298000 -0.940018000 0.000394000

8 2.264072000 -2.067327000 0.000861000

7 1.884199000 0.177983000 0.000400000

1 0.855503000 -0.002733000 0.000650000

6 2.261412000 1.494461000 -0.000046000

8 1.475595000 2.436760000 -0.000001000

1 3.932863000 2.652893000 -0.000947000

**TS^A-·T+^_A·T(rWC)↔A*·T(wWC)_**

7 -4.784734000 -0.554427000 -0.219360000

6 -4.883360000 -0.337608000 1.139177000

1 -5.817305000 -0.452859000 1.668235000

7 -3.740059000 0.012632000 1.675214000

6 -2.847591000 0.025080000 0.623646000

6 -1.438163000 0.339924000 0.595903000

7 -0.653988000 0.705955000 1.554295000

1 -1.177650000 0.739770000 2.426844000

1 0.167478000 1.699012000 -0.745332000

7 -0.883421000 0.233192000 -0.706175000

6 -1.640079000 -0.139770000 -1.771643000

1 -1.101158000 -0.208908000 -2.714460000

7 -2.920399000 -0.423684000 -1.799721000

6 -3.474242000 -0.323526000 -0.560909000

1 -5.517640000 -0.835292000 -0.852419000

7 3.154711000 1.674273000 0.036978000

6 4.139053000 0.748951000 0.368085000

1 5.079380000 1.176012000 0.690523000

6 3.914434000 -0.576340000 0.286046000

6 4.939388000 -1.610970000 0.636019000

1 5.877322000 -1.151130000 0.952363000

1 4.571268000 -2.254229000 1.439458000

1 5.133986000 -2.262038000 -0.220183000

6 2.587204000 -1.047338000 -0.162836000

8 2.282502000 -2.214810000 -0.271151000

7 1.667639000 -0.025491000 -0.486878000

1 0.506513000 -0.145234000 -0.651919000

6 1.939484000 1.257403000 -0.360236000

8 1.052801000 2.171802000 -0.639572000

1 3.300362000 2.666978000 0.152677000

**A*·T(w_WC_)**

7 5.256207000 -0.295438000 0.001056000

6 4.980365000 -1.645999000 0.002929000

1 5.764395000 -2.387927000 0.003977000

7 3.694395000 -1.896475000 0.003817000

6 3.097345000 -0.656674000 0.001779000

6 1.701127000 -0.302897000 0.001193000

7 0.648481000 -1.045002000 0.002391000

1 0.899349000 -2.030545000 0.004083000

1 0.579631000 1.447399000 -0.000604000

7 1.548251000 1.096203000 -0.000916000

6 2.569109000 1.991302000 -0.002448000

1 2.266291000 3.032793000 -0.004016000

7 3.840580000 1.698661000 -0.002227000

6 4.047403000 0.353984000 -0.000026000

1 6.162736000 0.147114000 0.000343000

7 -3.417370000 1.730666000 0.004496000

6 -4.546075000 0.934010000 0.006380000

1 -5.487624000 1.469683000 0.011543000

6 -4.473621000 -0.411476000 0.002232000

6 -5.672534000 -1.311729000 0.004171000

1 -6.599433000 -0.734401000 0.009530000

1 -5.669285000 -1.962912000 -0.874060000

1 -5.662467000 -1.968214000 0.878393000

6 -3.140111000 -1.034348000 -0.004765000

8 -2.950612000 -2.237044000 -0.009037000

7 -2.056867000 -0.138936000 -0.006412000

1 -1.086988000 -0.546028000 -0.009041000

6 -2.132984000 1.223772000 -0.001868000

8 -1.156136000 1.975661000 -0.003516000

1 -3.488503000 2.736668000 0.007367000

**TS_A*·T(wWC)↔A·T*O2(wWC)_**

7 5.110380000 -0.318106000 0.000477000

6 4.812992000 -1.665450000 0.000026000

1 5.586537000 -2.418428000 0.000047000

7 3.525339000 -1.899860000 -0.000421000

6 2.948003000 -0.650369000 -0.000259000

6 1.574841000 -0.263652000 -0.000551000

7 0.523246000 -1.032392000 -0.001109000

1 0.707945000 -2.027992000 -0.001191000

1 0.423505000 1.494160000 -0.000298000

7 1.414205000 1.113713000 -0.000220000

6 2.446397000 1.996332000 0.000365000

1 2.152777000 3.039849000 0.000616000

7 3.716888000 1.689951000 0.000647000

6 3.913555000 0.350063000 0.000305000

1 6.024639000 0.109135000 0.000881000

7 -3.336609000 1.724939000 -0.000478000

6 -4.439391000 0.894900000 -0.000155000

1 -5.400230000 1.396009000 -0.000287000

6 -4.309297000 -0.446449000 0.000198000

6 -5.470869000 -1.394093000 0.000482000

1 -6.422012000 -0.856150000 0.000107000

1 -5.436803000 -2.048857000 -0.874736000

1 -5.437054000 -2.048050000 0.876318000

6 -2.942547000 -1.004820000 0.000292000

8 -2.726711000 -2.213511000 0.000497000

7 -1.885530000 -0.100383000 0.000134000

1 -0.627009000 -0.613687000 -0.000483000

6 -2.043791000 1.233359000 -0.000155000

8 -1.092500000 2.051203000 -0.000130000

1 -3.433396000 2.728525000 -0.000404000

**A·T*_O2_(w_WC_)**

7 -5.201465000 -0.112059000 0.000083000

6 -5.027196000 -1.481785000 0.000089000

1 -5.867992000 -2.159373000 0.000108000

7 -3.768837000 -1.837710000 0.000069000

6 -3.075512000 -0.642542000 0.000049000

6 -1.689854000 -0.356899000 0.000024000

7 -0.746778000 -1.299310000 0.000017000

1 -1.022658000 -2.268139000 0.000022000

1 0.176989000 1.495542000 -0.000012000

7 -1.337238000 0.955929000 0.000008000

6 -2.294101000 1.904956000 0.000017000

1 -1.928348000 2.927371000 0.000002000

7 -3.609357000 1.744672000 0.000040000

6 -3.945217000 0.445881000 0.000056000

1 -6.073197000 0.395338000 0.000096000

7 3.326354000 1.733100000 -0.000046000

6 4.474439000 0.961201000 -0.000055000

1 5.407031000 1.511617000 -0.000060000

6 4.401353000 -0.384461000 -0.000057000

6 5.604702000 -1.277229000 -0.000067000

1 6.531773000 -0.699289000 -0.000073000

1 5.596086000 -1.932555000 0.875122000

1 5.596074000 -1.932552000 -0.875258000

6 3.059668000 -1.009193000 -0.000051000

8 2.911299000 -2.224256000 -0.000055000

7 1.949035000 -0.159394000 -0.000040000

1 0.249123000 -1.041846000 -0.000007000

6 2.099619000 1.132809000 -0.000037000

8 1.093833000 1.977996000 -0.000025000

1 3.364664000 2.740946000 -0.000037000

**А·Т(Н)**

7 2.909262000 2.111298000 0.000009000

6 1.538118000 2.052826000 -0.000004000

1 0.909493000 2.931457000 -0.000005000

7 1.090145000 0.820362000 -0.000011000

6 2.222387000 0.021168000 -0.000004000

6 2.421412000 -1.379337000 -0.000009000

7 1.415914000 -2.272087000 -0.000027000

1 1.662304000 -3.248731000 -0.000018000

1 0.433560000 -2.006925000 -0.000023000

7 3.691424000 -1.831598000 0.000001000

6 4.697860000 -0.950106000 0.000014000

1 5.694723000 -1.380835000 0.000021000

7 4.637706000 0.384109000 0.000017000

6 3.372868000 0.810889000 0.000008000

1 3.483237000 2.940510000 0.000007000

7 -3.818471000 1.464134000 0.000000000

6 -4.428133000 0.229004000 0.000007000

1 -5.511383000 0.249470000 0.000011000

6 -3.728360000 -0.926179000 0.000008000

6 -4.362267000 -2.285599000 0.000017000

1 -5.451618000 -2.210297000 -0.000013000

1 -4.052275000 -2.860249000 -0.876943000

1 -4.052323000 -2.860217000 0.877016000

6 -2.266657000 -0.834252000 -0.000002000

8 -1.521099000 -1.811333000 -0.000006000

7 -1.739495000 0.452469000 -0.000004000

1 -0.700397000 0.540027000 -0.000009000

6 -2.440025000 1.638643000 -0.000005000

8 -1.918940000 2.740041000 -0.000009000

1 -4.363614000 2.312991000 0.000003000

**TS^A-·T+^_А·Т(Н)↔A*N7·T(rwH)_**

7 3.261269000 1.448939000 1.135855000

6 1.969516000 1.659449000 1.524166000

1 1.685465000 2.450799000 2.199913000

7 1.144674000 0.792678000 0.959207000

6 1.945876000 -0.017309000 0.161813000

6 1.611042000 -1.153350000 -0.656226000

7 0.378588000 -1.588052000 -0.733687000

1 0.368210000 -2.406074000 -1.342677000

1 -0.313207000 -0.679850000 1.496228000

7 2.697535000 -1.717590000 -1.304857000

6 3.912684000 -1.217388000 -1.122151000

1 4.709327000 -1.719758000 -1.667654000

7 4.320181000 -0.180649000 -0.359506000

6 3.271066000 0.372686000 0.258021000

1 4.072181000 1.975188000 1.423352000

7 -3.387279000 1.017733000 -1.188758000

6 -3.939565000 -0.061575000 -0.568816000

1 -4.931118000 -0.340281000 -0.903777000

6 -3.285653000 -0.756263000 0.402424000

6 -3.838184000 -1.971955000 1.089628000

1 -4.837949000 -2.201521000 0.718009000

1 -3.194178000 -2.837258000 0.916406000

1 -3.898737000 -1.822596000 2.170489000

6 -1.969375000 -0.292382000 0.694630000

8 -1.266593000 -0.912160000 1.593924000

7 -1.468645000 0.792937000 0.090437000

1 -0.458813000 1.018502000 0.330509000

6 -2.114047000 1.521085000 -0.905297000

8 -1.648298000 2.486565000 -1.454179000

1 -3.886484000 1.507660000 -1.918740000

**А*_N7_·Т(rw_Н_)**

7 3.571760000 1.975752000 -0.000010000

6 2.285855000 2.391760000 0.000003000

1 1.961796000 3.417908000 -0.000003000

7 1.490192000 1.331466000 0.000008000

6 2.267046000 0.187447000 -0.000001000

6 1.948094000 -1.215892000 0.000011000

7 0.719461000 -1.649033000 0.000024000

1 0.706307000 -2.667594000 0.000029000

1 0.436795000 1.359117000 0.000012000

7 3.065539000 -2.031086000 0.000005000

6 4.280334000 -1.507734000 -0.000006000

1 5.098641000 -2.223706000 -0.000010000

7 4.664550000 -0.209887000 -0.000014000

6 3.596709000 0.581625000 -0.000010000

1 4.388297000 2.569001000 -0.000006000

7 -4.090479000 -1.212743000 -0.000016000

6 -4.429300000 0.115050000 -0.000010000

1 -5.492066000 0.326444000 -0.000015000

6 -3.496392000 1.093772000 0.000000000

6 -3.826738000 2.557468000 0.000004000

1 -4.907651000 2.713042000 0.000002000

1 -3.404944000 3.054767000 0.877696000

1 -3.404940000 3.054772000 -0.877683000

6 -2.095971000 0.682956000 0.000004000

8 -1.173690000 1.520134000 0.000008000

7 -1.828232000 -0.664776000 0.000004000

1 -0.793813000 -0.996632000 0.000012000

6 -2.772913000 -1.675274000 -0.000005000

8 -2.511385000 -2.860054000 -0.000003000

1 -4.797705000 -1.932658000 -0.000018000

**TS_А*N7·Т(rwН)↔А·Т*(rwН)_**

7 -3.526400000 1.976600000 0.000019000

6 -2.242246000 2.399525000 0.000092000

1 -1.927658000 3.429057000 0.000001000

7 -1.434980000 1.350981000 0.000131000

6 -2.205556000 0.201312000 0.000084000

6 -1.885804000 -1.197502000 0.000121000

7 -0.654883000 -1.641071000 0.000100000

1 -0.615982000 -2.656893000 0.000220000

1 -0.360043000 1.391405000 -0.000069000

7 -2.986870000 -2.021881000 0.000174000

6 -4.209298000 -1.509616000 0.000115000

1 -5.019752000 -2.233839000 0.000086000

7 -4.600147000 -0.217525000 0.000032000

6 -3.539216000 0.583513000 0.000034000

1 -4.347198000 2.564041000 0.000107000

7 3.997687000 -1.246622000 0.000408000

6 4.373773000 0.069568000 0.000516000

1 5.441603000 0.254585000 0.000933000

6 3.462673000 1.068917000 0.000078000

6 3.827198000 2.524567000 0.000231000

1 4.911738000 2.654914000 -0.000093000

1 3.416912000 3.032372000 -0.877007000

1 3.417512000 3.032080000 0.877932000

6 2.054859000 0.682512000 -0.000434000

8 1.158996000 1.562305000 -0.000653000

7 1.737530000 -0.643877000 -0.000715000

1 0.591129000 -1.025492000 -0.000434000

6 2.662811000 -1.665364000 -0.000230000

8 2.377297000 -2.849224000 -0.000239000

1 4.682676000 -1.987716000 0.000776000

**А·Т*(rw_Н_)**

7 3.359375000 2.104640000 -0.000010000

6 2.011425000 2.342841000 -0.000014000

1 1.600684000 3.341033000 -0.000020000

7 1.305087000 1.236315000 -0.000009000

6 2.239698000 0.207980000 -0.000001000

6 2.139315000 -1.207792000 0.000006000

7 0.973772000 -1.863298000 0.000007000

1 0.989413000 -2.871013000 0.000008000

1 -0.399253000 1.347128000 0.000001000

7 3.291062000 -1.913118000 0.000011000

6 4.457898000 -1.263310000 0.000009000

1 5.342866000 -1.892906000 0.000014000

7 4.679586000 0.054727000 0.000003000

6 3.531437000 0.734430000 -0.000002000

1 4.100079000 2.789152000 -0.000013000

7 -3.974520000 -1.364482000 -0.000006000

6 -4.453678000 -0.089091000 0.000001000

1 -5.531866000 0.019199000 0.000002000

6 -3.611683000 0.975792000 0.000006000

6 -4.071161000 2.405524000 0.000013000

1 -5.162029000 2.461112000 0.000014000

1 -3.699766000 2.940452000 0.878284000

1 -3.699766000 2.940462000 -0.878252000

6 -2.209151000 0.633728000 0.000003000

8 -1.365126000 1.647758000 0.000007000

7 -1.746024000 -0.596958000 -0.000004000

1 0.070620000 -1.387052000 0.000001000

6 -2.600411000 -1.674203000 -0.000008000

8 -2.238751000 -2.839076000 -0.000014000

1 -4.600463000 -2.157194000 -0.000009000

**А·Т(rН)**

7 -2.544920000 -2.323558000 0.000012000

6 -1.209223000 -2.008189000 0.000015000

1 -0.425712000 -2.752126000 0.000023000

7 -1.001277000 -0.713515000 0.000011000

6 -2.263577000 -0.141538000 0.000000000

6 -2.722929000 1.196041000 -0.000009000

7 -1.904464000 2.263719000 -0.000012000

1 -2.331719000 3.175792000 -0.000005000

1 -0.890724000 2.190006000 0.000007000

7 -4.055052000 1.401636000 -0.000016000

6 -4.877848000 0.346527000 -0.000014000

1 -5.937933000 0.581989000 -0.000020000

7 -4.567552000 -0.952359000 -0.000007000

6 -3.245041000 -1.133781000 0.000000000

1 -2.952264000 -3.246131000 0.000001000

7 3.305986000 1.832006000 -0.000014000

6 4.314595000 0.889953000 -0.000013000

1 5.320085000 1.293117000 -0.000022000

6 4.066931000 -0.436509000 -0.000010000

6 5.139768000 -1.484336000 -0.000018000

1 6.133040000 -1.030659000 -0.000027000

1 5.050916000 -2.131558000 0.876699000

1 5.050901000 -2.131558000 -0.876734000

6 2.665067000 -0.873342000 0.000002000

8 2.306249000 -2.041707000 -0.000001000

7 1.717391000 0.156509000 0.000019000

1 0.714589000 -0.128488000 0.000024000

6 1.961203000 1.504358000 0.000014000

8 1.084929000 2.361027000 0.000036000

1 3.514923000 2.818882000 0.000018000

**TS_А·Т(rН)↔А*N7·Т(wН)_**

7 -3.304822000 1.768082000 0.342549000

6 -2.127835000 2.039099000 0.980630000

1 -1.943223000 2.972864000 1.488507000

7 -1.272543000 1.038554000 0.876486000

6 -1.929185000 0.065970000 0.126200000

6 -1.523577000 -1.254395000 -0.278165000

7 -0.356113000 -1.753103000 0.062441000

1 -0.299465000 -2.700459000 -0.310397000

1 -0.063425000 -0.475153000 2.102659000

7 -2.469800000 -1.935115000 -1.020232000

6 -3.643924000 -1.371967000 -1.279989000

1 -4.330340000 -1.977352000 -1.868613000

7 -4.119898000 -0.163366000 -0.922116000

6 -3.200293000 0.502211000 -0.215431000

1 -4.110123000 2.371302000 0.274735000

7 2.749301000 -1.110110000 1.209287000

6 3.647891000 -0.873433000 0.174873000

1 4.495441000 -1.544816000 0.144105000

6 3.456310000 0.120258000 -0.713267000

6 4.385291000 0.408302000 -1.851654000

1 5.233478000 -0.277979000 -1.855703000

1 3.854951000 0.321742000 -2.803437000

1 4.757828000 1.434189000 -1.792367000

6 2.259837000 0.969199000 -0.576393000

8 1.973729000 1.889287000 -1.307076000

7 1.435069000 0.651458000 0.532625000

1 0.469357000 1.075265000 0.608886000

6 1.632527000 -0.385664000 1.334806000

8 0.867686000 -0.647748000 2.361188000

1 2.833589000 -1.924208000 1.801787000

**А*_N7_·Т(w_Н_)**

7 3.943772000 1.619216000 -0.000019000

6 2.752954000 2.258072000 -0.000012000

1 2.620801000 3.326390000 -0.000015000

7 1.776579000 1.364465000 0.000000000

6 2.335653000 0.097724000 0.000001000

6 1.781259000 -1.226475000 0.000014000

7 0.493591000 -1.456570000 0.000024000

1 0.280419000 -2.450476000 0.000030000

1 0.731236000 1.593007000 0.000008000

7 2.725528000 -2.226596000 0.000013000

6 4.017332000 -1.931792000 0.000001000

1 4.691772000 -2.784088000 0.000001000

7 4.623539000 -0.725552000 -0.000011000

6 3.715574000 0.244798000 -0.000010000

1 4.854178000 2.055151000 -0.000028000

7 -3.009088000 1.837857000 0.000010000

6 -4.170230000 1.089795000 0.000001000

1 -5.090848000 1.660875000 0.000002000

6 -4.138777000 -0.256645000 -0.000008000

6 -5.364367000 -1.119507000 -0.000019000

1 -6.274425000 -0.515346000 -0.000011000

1 -5.376096000 -1.774064000 -0.875743000

1 -5.376096000 -1.774086000 0.875690000

6 -2.819742000 -0.915502000 -0.000007000

8 -2.686693000 -2.130786000 -0.000011000

7 -1.695815000 -0.079224000 0.000000000

1 -0.622569000 -0.654680000 0.000011000

6 -1.755168000 1.263617000 0.000011000

8 -0.755466000 2.019765000 0.000022000

1 -3.034767000 2.845817000 0.000018000

**TS_А*N7·Т(wН)↔А·Т*O2(wН)_**

7 3.943772000 1.619216000 -0.000019000

6 2.752954000 2.258072000 -0.000012000

1 2.620801000 3.326390000 -0.000015000

7 1.776579000 1.364465000 0.000000000

6 2.335653000 0.097724000 0.000001000

6 1.781259000 -1.226475000 0.000014000

7 0.493591000 -1.456570000 0.000024000

1 0.280419000 -2.450476000 0.000030000

1 0.731236000 1.593007000 0.000008000

7 2.725528000 -2.226596000 0.000013000

6 4.017332000 -1.931792000 0.000001000

1 4.691772000 -2.784088000 0.000001000

7 4.623539000 -0.725552000 -0.000011000

6 3.715574000 0.244798000 -0.000010000

1 4.854178000 2.055151000 -0.000028000

7 -3.009088000 1.837857000 0.000010000

6 -4.170230000 1.089795000 0.000001000

1 -5.090848000 1.660875000 0.000002000

6 -4.138777000 -0.256645000 -0.000008000

6 -5.364367000 -1.119507000 -0.000019000

1 -6.274425000 -0.515346000 -0.000011000

1 -5.376096000 -1.774064000 -0.875743000

1 -5.376096000 -1.774086000 0.875690000

6 -2.819742000 -0.915502000 -0.000007000

8 -2.686693000 -2.130786000 -0.000011000

7 -1.695815000 -0.079224000 0.000000000

1 -0.622569000 -0.654680000 0.000011000

6 -1.755168000 1.263617000 0.000011000

8 -0.755466000 2.019765000 0.000022000

1 -3.034767000 2.845817000 0.000018000

**А·Т*_O2_(w_H_)**

7 3.772435000 1.768194000 -0.000023000

6 2.488650000 2.238721000 -0.000021000

1 2.257967000 3.293099000 -0.000029000

7 1.599927000 1.271753000 -0.000010000

6 2.339208000 0.093999000 -0.000004000

6 1.995708000 -1.284516000 0.000002000

7 0.737595000 -1.733912000 0.000014000

1 0.586143000 -2.730532000 0.000018000

1 -0.006614000 1.677315000 0.000044000

7 3.010891000 -2.176392000 0.000000000

6 4.271630000 -1.738057000 -0.000008000

1 5.034982000 -2.510585000 -0.000010000

7 4.716987000 -0.477554000 -0.000014000

6 3.702782000 0.388706000 -0.000012000

1 4.621645000 2.312372000 -0.000036000

7 -3.145414000 1.807989000 -0.000013000

6 -4.268415000 0.999601000 -0.000037000

1 -5.218129000 1.519693000 -0.000070000

6 -4.151464000 -0.342926000 -0.000022000

6 -5.324742000 -1.274748000 -0.000049000

1 -6.270259000 -0.727637000 -0.000075000

1 -5.294381000 -1.929320000 -0.875266000

1 -5.294426000 -1.929313000 0.875175000

6 -2.790370000 -0.922821000 0.000021000

8 -2.601464000 -2.132390000 0.000027000

7 -1.705946000 -0.037954000 0.000056000

1 -0.077389000 -1.116227000 0.000024000

6 -1.902035000 1.245240000 0.000036000

8 -0.922757000 2.126527000 0.000066000

1 -3.216816000 2.813990000 -0.000023000
